# Alterations in choroid plexus gene expression in Alzheimer’s disease provide inferences for CSF composition and dynamics

**DOI:** 10.1186/1743-8454-7-S1-S48

**Published:** 2010-12-15

**Authors:** Miles C Miller, Edward G Stopa, Elena V Nikonova, Keith Q Tanis, Alexei A Podtelezhnikov, Eva M Finney, David J Stone, Luiz M Camargo, Lisan Parker, Ajay Verma, Andrew Baird, John E Donahue, Ana Maria Gonzalez, Brian Eliceiri, Gerald D Silverberg, Petra M Klinge, Conrad E Johanson

**Affiliations:** 1 Departments of Neurosurgery and Pathology (Neuropathology Division), Rhode Island Hospital, The Warren Alpert Medical School, Brown University, Providence, RI, USA; 2 Molecular Profiling & Research Informatics, Merck Sharp & Dohme, West Point, PA, USA; 3 Department of Surgery, University of California San Diego Medical Center, Hillcrest, 212 Dickinson Street, San Diego, CA, USA; 4 School of Clinical and Experimental Medicine, College of Medical and Dental Sciences, University of Birmingham, Edgbaston, UK

## Background

Alterations in CSF composition are associated with normal aging and the pathophysiology of Alzheimer’s disease and NPH. These changes may contribute to modified choroid plexus function in these states. Disease-related changes in choroid plexus gene expression were investigated using human Affymetrix 48K gene arrays.

## Materials and methods

Lateral ventricle choroid plexuses were excised at autopsy from healthy aged controls (mean age/mean PMI: 58 years/22 hours) and patients with advanced (Braak & Braak stage V-VI) Alzheimer’s disease (79/18). Diseased control patients with frontotemporal dementia (72/NA) and Huntington’s disease (71/19) were also sampled. All tissue specimens were snap frozen in liquid nitrogen and stored at -80°C until use. RNA was extracted from choroid plexuses with Trizol followed by NuGEN Ovation amplification, and cDNA was hybridized to custom chips at Rosetta/Merck. RMA normalization was performed, and data were analyzed using one way ANOVA to identify the gene sets of greatest significance. These sets were then analyzed further for biological enrichment using individual (Ingenuity) and combined (Target and Gene Information System) pathway tools.

## Results

Differences on the level of gene expression were seen in the choroid plexuses of Braak & Braak V-VI AD patients when compared to both the normal and diseased (FTD, HD) control groups. Four experimental groups could significantly be separated out based on the analyses of 648 sequences (p<0.001, FDR~8%), with close to fifty percent of those sequences being up-regulated in neurodegenerative disease states. There was a significant increase in immune response noted in advanced AD patients, while amyloid processing and oxidative phosphorylation were both down-regulated in these cases. In addition, cellular adhesion function and extracellular matrix re-modeling were highly enriched in the advanced AD patients when compared to both the healthy and diseased controls. Other important observations that were made in analyzing these data include: decreases in PPARa/RXRa nuclear receptor/retinoic acid, a-adrenergic, glucocorticoid and melatonin signaling, as well as N-glycan, glutathione (antioxidant) and ubiquinone metabolism in AD choroid plexuses.

## Conclusions

These gene expression profiles may serve as valuable resources to investigators working in the fields of aging CSF dynamics and hydrocephalus. It is readily available and can be shared with interested investigators to address specific questions pertaining to their area of investigation.

